# Platyconic Acid A, Platycodi Radix-Derived Saponin, Suppresses TGF-β1-Induced Activation of Hepatic Stellate Cells via Blocking SMAD and Activating the PPARγ Signaling Pathway

**DOI:** 10.3390/cells8121544

**Published:** 2019-11-29

**Authors:** Jae Ho Choi, Seul Mi Kim, Gi Ho Lee, Sun Woo Jin, Hyun Sun Lee, Young Chul Chung, Hye Gwang Jeong

**Affiliations:** 1 Department of Toxicology, College of Pharmacy, Chungnam National University, Daejeon 34134, Korea; choijh1@cnu.ac.kr (J.H.C.); sarah5350@naver.com (S.M.K.); ghk1900@cnu.ac.kr (G.H.L.); mpassword@cnu.ac.kr (S.W.J.); 2 Natural Medicine Research Center, Korea Research Institute of Bioscience and Biotechnology, Ochang 28116, Korea; leehs@kribb.re.kr; 3 Department of Food and Medicine, College of Public Health and Natural Science, International University of Korea, Jinju 52833, Korea; fnjung@hanmil.net

**Keywords:** platyconic acid A, TGF-β1, hepatic stellate cells, SMAD, PPARγ

## Abstract

Platycodi radix is a widely sold health food worldwide, which contains numerous phytochemicals that are beneficial to health. Previously, we reported that saponin from the roots of Platycodi radix-derived saponin inhibited toxicant-induced liver diseases. Nevertheless, the inhibitory effect of platyconic acid A (PA), the active component of Platycodi radix-derived saponin, on the anti-fibrotic activity involving the SMAD pathway remains unclear. We investigated the inhibitory effects of PA on TGF-β1-induced activation of hepatic stellate cells (HSCs). PA inhibited TGF-β1-enhanced cell proliferation, as well as expression of α-SMA and collagen Iα1 in HSC-T6 cells. PA suppressed TGF-β1-induced smad2/3 phosphorylation and smad binding elements 4 (SBE4) luciferase activity. Reversely, PA restored TGF-β1-reduced expression of smad7 and peroxisome proliferator-activated receptor (PPAR)γ. PA also repressed TGF-β1-induced phosphorylation of Akt and MAPKs. In summary, the results suggest that the inhibitory effect of PA on HSCs occurs through the blocking of SMAD-dependent and SMAD-independent pathways, leading to the suppression of α-SMA and collagen Iα1 expression.

## 1. Introduction

Liver diseases constitute one of the world’s major health problems, causing serious morbidity and mortality [1]. Hepatic fibrosis can progress to liver cirrhosis accompanied by severe liver disease, leading to disorders of normal liver architecture and nodule formation, liver cancer, and ultimately liver failure. Hepatic fibrosis is generally prognostic for chronic liver injury caused by alcoholic abuse, obesity, and viral infection [2]. Although various risk factors have been reported and disease progress has been well studied, the mechanisms of therapeutic targets for various forms of chronic liver disease remain unclear.

Hepatic stellate cells (HSCs), a nonparenchymal cell type within the perisinusoidal space of Disse, contribute to key homeostatic functions of the liver, including regeneration, immunoregulation, drug metabolism, and detoxification. In normal liver, HSCs are stored as non-proliferative, quiescent cells with lipid droplets containing retinol [3]. When HSCs are activated, they lose their lipid droplets and differentiate into activated myoblast cells, which express smooth muscle actin and various types of collagen proteins, resulting in extracellular matrix deposition and fibrosis during chronic liver injury [4]. After their activation, growth factors cause the proliferation of HSCs.

Transforming growth factor (TGF)-β is a major regulator of physiological and pathological fibrosis. It induces proliferation and migration of HSCs in the liver and is generally well-known as an important therapeutic treatment for fibrotic diseases [5]. The activation of HSCs by the TGF-β signal pathway is related to SMAD-dependent and SMAD-independent pathways. Intracellular TGF-β signaling is mediated mainly through SMAD-dependent pathways. TGF-β signaling is initiated by a ligand that binds to the type 2 TGF-β receptor (TβRII), which induces type 1 TGF-β receptor (TβRI) phosphorylation to induce smad2 and smad3 phosphorylation, followed by binding to smad4 to form a complex. The SMAD complex binds to smad binding elements (SBEs) of the gene promoter, followed by translocation to the nucleus where it regulates transcription of the target gene. In contrast, smad7, a negative regulator of the TGF-β1 signaling pathway, binds to TβRI to block the phosphorylation of smad2/3 [6]. TGFβ also activates Akt and the mitogen-activated protein kinases (MAPKs) signal pathways, including ERK1/2, JNK1/2, and p38 MAPK, via a SMAD-independent pathway to promote HSCs activation [7,8].

Peroxisome proliferator-activated receptor (PPAR)γ is a ligand-dependent nuclear receptor, which modulates the negative role of the profibrotic signaling pathway involving fibrogenesis in liver tissue [9,10]. PPARγ blocks the TGF-β pathway to suppress myofibroblast transdifferentiation from fibroblasts. Finally, PPARγ inhibits TGF-β-induced downstream signal transduction and hepatic fibrogenesis [11]. Recent studies have reported that PPARγ is activated by natural and pharmacological agents. These PPARγ agonists prevent TGF-β-induced smad2/3 phosphorylation, nuclear accumulation, and recruitment of the smad complex [12]. Therefore, HSCs modulation by SMAD and PPAR-γ represents the most relevant therapeutic targets for possible drug treatments of liver fibrosis.

*Platycodon grandiflorum* A. DC (Campanulaceae) is mainly distributed in Northeast Asia, and has been used as a food resource in the Asian countries of the Korea, Japan, and China [13]. Platycodi radix, the root of *P. grandiflorum*, is used as in traditional medicine (Chinese name: “Jiegeng;” Japanese name: “Kikyo;” Korean name: “Doraji”) [14]. Recently, Platycodi radix, which contains various nutrients (amino acids, trace elements, and unsaturated fatty acids such as linoleic acid), has been considered to be a food source [15]. Our previous studies have reported that aqueous extract and saponin derived from Platycodi radix has protective effects on atopic dermatitis-like skin diseases, airway inflammation, osteoporosis, and metastasis [16,17,18,19,20,21,22]. Importantly, we reported that an aqueous extract from Platycodi radix and Platycodi radix-derived saponin inhibited various hepatic toxicant-induced liver diseases via numerous biological and pharmacological effects. For example, Platycodi radix inhibited carbon tetrachloride- and dimethylnitrosamine-induced hepatoxicity, hepatic inflammatory, and fibrogenic markers, including TNF-α, COX-2, NF-κB, MMP-13, TIMP-1, α-SMA, and collagen type I via induction of Nrf2-mediated antioxidant enzymes, including γ-GCS, HO-1, NQO1, and GST [23,24]. Platycodi radix-derived saponin also ameliorates high-fat diet-induced nonalcoholic fatty liver diseases and nonalcoholic steatohepatitis symptoms, including hepatic inflammation and fibrogenesis via induction of Nrf2-mediated antioxidant enzymes [25,26]. Notably, Platycodi radix-derived saponin increases the PPARα-regulated fatty acid oxidation markers acyl-CoA oxidase and carnitine-palmitoyl-CoA transferase-1. These results suggest that Platycodi radix-derived saponin reduces hepatic toxicant-induced chronic damage via modulation of antioxidant enzymes.

However, the inhibitory effect of platyconic acid A (PA), the active component of Platycodi radix-derived saponin, on their anti-fibrotic effect remains unclear. Therefore, the objective of this study was to characterize the inhibitory effect of PA on TGF-β1-induced activation of HSCs involving the SMAD and PPARγ signal transduction pathways. The results showed that PA decreased hepatic fibrogenesis by blocking SMAD and activating PPARγ, suggesting that PA could be developed as an alternative treatment for patients with chronic liver fibrosis.

## 2. Materials and Methods

### 2.1. Chemicals and Reagents

The MTT assay kit was purchased from Abcam (ab211091; Cambridge, MA, USA), and the LDH cytotoxicity detection kit was purchased from Roche (04 744 926 001; Mannheim, Germany). The WST-1 cell proliferation assay kit (MK400) and RNAiso reagent (9109) were purchased from Takara Bio (Kusatsu, Japan). LY294002, PD98059, SB203580, and SP600125 were purchased from Calbiochem (La Jolla, CA, USA). GW9662 was purchased from Cayman Chemical (Ann Arbor, MI, USA). Lipofectamine™ 2000 transfection reagent was purchased from Invitrogen (11668019; Carlsbad, CA, USA). TGF-β1 was purchased from R&D systems (240-B; Minneapolis, MN, USA). Ethanol (100983) and DMSO (101900) were purchased from Merck (Darmstadt, Germany). Antibodies to the following components were purchased from the following sources: α-SMA (M0851; Agilent, CA, USA); collagen Iα1 (NB600-408; Novus Biologicals, CO, USA); PPARγ (ABN1445; Merck, Darmstadt, Germany); smad2 (sc-393312), smad3 (sc-101154), smad7 (sc-365846), and β-actin (sc-47778) (Santa Cruz Biotechnology, CA, USA); p-smad2 (3108), p-smad3 (9520), p-Akt (9271), p-ERK1/2 (9101), p-JNK1/2 (9251), p-p38 MAPK (9211), Akt (9272), ERK1/2 (9102), JNK1/2 (9252), p38 MAPK (9212), and secondary antibodies coupled with horseradish peroxidase (HRP) (anti-rabbit (7404) or anti-mouse (7076) IgG; Cell Signaling Technology, MA, USA). The enhanced chemiluminescence solution was purchased from BIOFACT (OP101-200; Daejeon, Republic of Korea). The Nitrocellulose Membranes membrane was purchased from Amersham Pharmacia Biotech (10600002; Piscataway, NJ, USA). Dulbecco’s Modified Eagle’s Medium (LM 001-05; DMEM; high glucose), fetal bovine serum (FBS; S 001-07), and penicillin-streptomycin (LS 202-02) solutions were purchased from WELGENE (Gyeongsan, Republic of Korea). All chemicals were of the highest commercially available grade.

### 2.2. Preparation of PA

**Figure 1:**
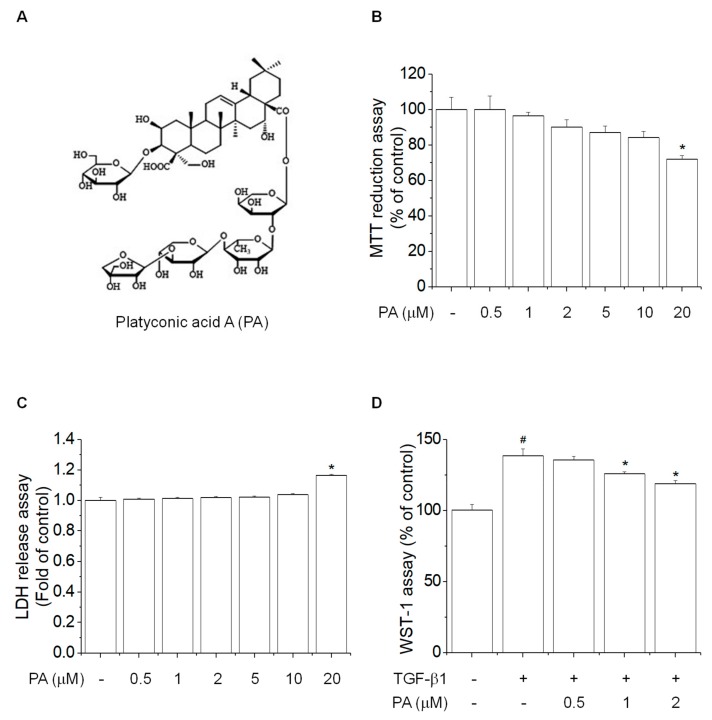
The effects of platyconic acid A (PA) on TGF-β1-induced cell proliferation in HSC-T6 cells. (**A**) The chemical structure of PA; (**B**,**C**) the effect of PA on viability and cytotoxicity in rat hepatic stellate cells (HSCs). Cells were treated with various concentrations of PA at 37 °C for 24 h, and cell viability was determined using the MTT assay, while cell cytotoxicity was analyzed using the LDH assay. The results are expressed as the means ± SD of three independent experiments. * Significantly different from the control (*p* < 0.01); (**D**) the inhibitory effect of PA on TGF-β1-induced cell proliferation in rat HSCs. Cells were pretreated with 0.5, 1, and 2 μM PA for 1 h, and then stimulated with TGF-β1 (5 ng/mL) for 24 h. Cell proliferation was determined using the WST-1 assay. The results are expressed as the means ± SD of three independent experiments. # Significantly different from the control (*p* < 0.01). * Significantly different from the TGF-β1-treated group (*p* < 0.01).

Our previous study reported platyconic acid A (PA) isolation and purification (>95%) from Platycodi radix-derived saponin [19,27]. The chemical structure of PA is shown in Figure 1A.

### 2.3. Cell Culture

HSC-T6 cells were cultured in high-glucose DMEM supplemented with 10% FBS and 1% penicillin-streptomycin solution. HSC-T6 cells were kept in a humidified atmosphere with 5% CO_2_ at 37 °C. Before drug treatment, the cells were changed to serum-free medium overnight. The cells were pretreated with PA for 1 h, treated with TGF-β1 (5 ng/mL) for 24 h, and then harvested for further assays. PA was dissolved in DMSO for all experiments. The final DMSO concentration never exceeded 0.1%, and the solvent had no noticeable effect on the assays.

### 2.4. Cell Viability Assay

The effects of PA on the viability, cytotoxicity, and proliferation of cells were assessed using the MTT, LDH, and WST-1 assay kits according to the manufacturers’ instructions.

### 2.5. Real Time-Polymerase Chain Reaction

Total RNA was extracted from PA-treated cells using RNAiso reagent according to the manufacturer’s protocol. Accumulated PCR products were detected directly by monitoring the increase in the reporter dye (SYBR; DQ383-40h) signal. The quantity of each transcription was calculated according to the manufacturer’s instructions and normalized to the amount of GAPDH as a housekeeping gene. The real time-PCR primer sequences are listed in Table 1.

### 2.6. Luciferase Assay

Cells were incubated in 24-well plates (5 × 10^5^ cells/well) overnight and transiently co-transfected with smad binding elements 4 (SBE4)-Luc (0.5 μg) and pCMV-β-gal (0.2 μg) using Lipofectamine™ 2000 reagent according to the manufacturer’s instructions. After 5 h, the transfection medium was replaced with the basal medium. The cells were pretreated with PA for 1 h and then treated with TGF-β1 for 24 h. Luciferase activity was measured using a luminometer (Luminoscan Ascent; Thermo Electron, Langenselbold, Germany), normalized to β-galactosidase plasmid (#45964; Addgene, Cambridge, MA, USA), and expressed relative to the luciferase activity of control cells. The SBE4-Luc promoter vector was obtained from Addgene (#16495; Cambridge, MA, USA).

### 2.7. Western Blotting

The cell lysates were separated by sodium dodecyl sulfate-polyacrylamide gel electrophoresis and transferred to NC membranes, and then incubated with the appropriate primary and HRP-conjugated secondary antibodies. Membranes were visualized using an enhanced chemiluminescence Western blotting detection kit. Protein bands were imaged using densitometry and analyzed using ImageJ software (National Institutes of Health, Bethesda, MD, USA). The relative expression levels of target proteins were normalized using β-actin as an internal control.

### 2.8. Statistical Analysis

All experiments were performed in triplicate. The results are expressed as the means ± standard deviation (SD). Statistical significance was determined using one-way analysis of variance followed by the Tukey-Kramer test, with a value of *p* < 0.01 indicating significance. A statistical software package (GraphPad Software, San Diego, CA, USA) was used for all statistical calculations. 

## 3. Results

### 3.1. PA Reduces TGF-β1-Induced HSCs Proliferation

To examine the inhibitory effects of platyconic acid A (PA) on rat HSCs activation, we examined the cell viability and cell cytotoxic effects of HSC-T6 cells following treatment with various PA concentrations for 24 h. The MTT and LDH assays showed no cytotoxic effects at concentrations <10 μM PA (Figure 1B,C). Then, we examined the inhibitory effect of PA on TGF-β1-induced cell proliferation using the WST-1 assay, which showed that PA suppressed TGF-β1-induced cell proliferation in a concentration-dependent manner (Figure 1D). Based on these results, we selected 0.5, 1, and 2 μM PA concentrations for the subsequent experiments.

### 3.2. PA Reduces TGF-β1-Induced HSCs Activation

**Figure 2:**
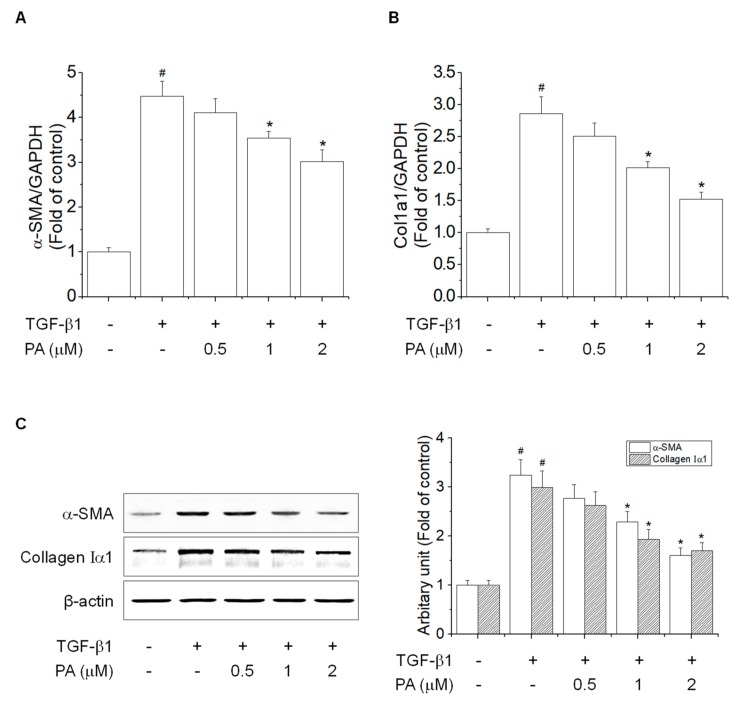
The effects of PA on TGF-β1-induced α- SMA and collagen Iα1 expression in HSC-T6 cells. (**A**,**B**) The inhibitory effect of PA on TGF-β1-induced α-SMA and collagen type I mRNA and protein expression in rat hepatic stellate cells. Cells were pretreated with 0.5, 1, and 2 μM PA for 1 h, and then stimulated with TGF-β1 (5 ng/mL) for 24 h. Total RNA extracted from cells was analyzed by the real-time polymerase chain reaction to determine α-SMA and ColIa1 mRNA expression; (**C**) the total protein extracted from cells was subjected to Western blotting to determine α-SMA and collagen Iα1 expression. Protein bands were imaged using densitometry and analyzed using ImageJ software. The relative expression levels of target proteins were normalized using β-actin as an internal control. The results are expressed as the means ± SD of three independent experiments. # Significantly different from the control (*p* < 0.01). * Significantly different from the TGF-β1-treated group (*p* < 0.01).

Typical features of HSCs activation involve the expression of α-SMA and collagen I by TGF-β1 [28]. We examined the effects of PA on TGF-β1-induced α-SMA and collagen Iα1 expression in HSC-T6 cells, which showed that PA inhibited TGF-β1-induced mRNA and protein expression of α-SMA and collagen Iα1 in a concentration-dependent manner (Figure 2). These results indicated that PA decreased the TGF-β1-induced activation of HSCs via inhibition of transcription and translation.

### 3.3. PA Reduces TGF-β1-Induced HSCs Activation by Blocking a SMAD-Dependent Signal Pathway

**Figure 3:**
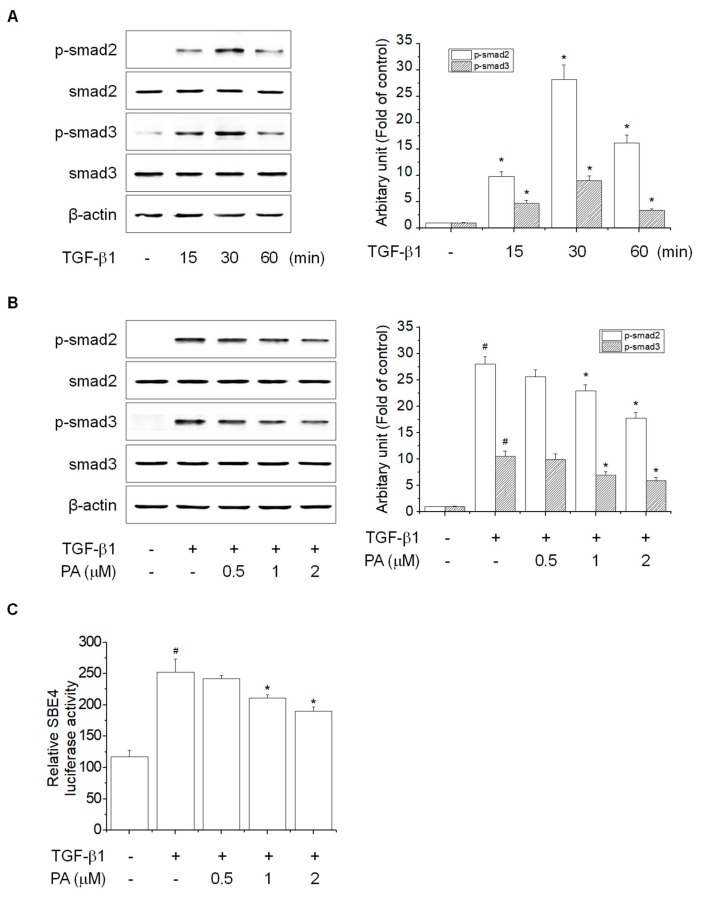
The effects of PA on the TGF-β1-induced SMAD signal pathway in HSC-T6 cells. (**A**) The effect of TGF-β1-induced smad2 and smad3 phosphorylation in rat hepatic stellate cells (HSCs). Cells were treated with TGF-β1 (5 ng/mL) for 0, 15, 30, and 60 min, and smad2 and smad3 phosphorylation was analyzed by Western blotting; (**B**) the inhibitory effect of PA on TGF-β1-induced smad2 and smad3 phosphorylation in rat HSCs. Cells were pretreated with 0.5, 1, and 2 μM PA for 1 h, and then stimulated with TGF-β1 (5 ng/mL) for 30 min. The total protein extracted from cells was subjected to Western blotting to assess smad2 and smad3 phosphorylation. Protein bands were imaged using densitometry and analyzed using ImageJ software. The relative phosphorylation levels of target proteins were normalized using β-actin as an internal control; (**C**) the inhibitory effect of PA on TGF-β1-induced SBE4 luciferase activity in rat HSCs. Cells were transiently transfected with luciferase reporter containing four copies of the smad binding element sites (SBE4), cultured with PA (0.5, 1, and 2 μM) and/or TGF-β1 (5 ng/mL) for 24 h, and the relative luciferase activity in the cell extract was determined. The results are expressed as the means ± SD of three independent experiments. # Significantly different from the control (*p* < 0.01). * Significantly different from the TGF-β1-treated group (*p* < 0.01).

TGF-β signal transduction involves TGF-β1 binding to type II TGF-β receptors (TβRII), followed by recruitment and activation of type I TGF-β receptors (TβRI) [29]. Activated TβRI phosphorylates smad2 and smad3, and then forms complexes with smad4, which translocates into the nucleus and binds to smad binding element (SBE) to activate HSCs [6]. To examine the effects of PA on TGF-β1-induced phosphorylation of smad2 and smad3, we first determined the timing of TGF-β1-induced phosphorylation of smad2 and smad3 in HSC-T6 cells. TGF-β1 induced a significant increase in smad2 and smad3 phosphorylation at 30 min (Figure 3A). PA decreased TGF-β1-induced phosphorylation of smad2 and smad3 in a concentration-dependent manner (Figure 3B), and PA decreased TGF-β1-induced SBE4 luciferase activity in a concentration-dependent manner (Figure 3C). Together, these results indicated that PA reduced TGF-β1-induced HSCs activation by suppression of SBE activity via smad2/3 phosphorylation.

**Figure 4:**
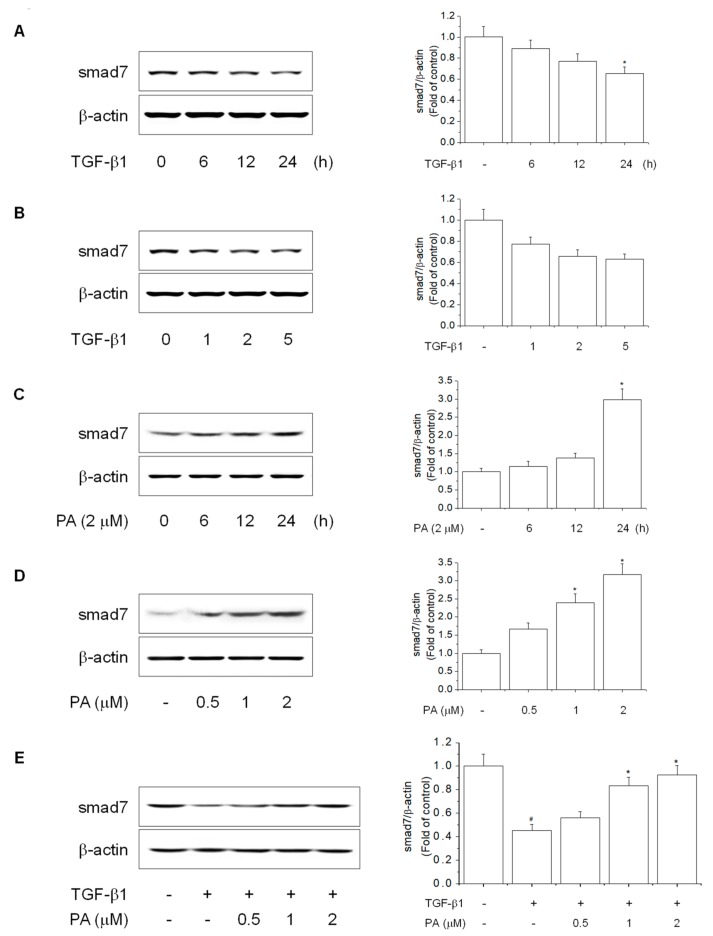
The effects of PA on TGF-β1-reduced smad7 expression in HSC-T6 cells. (**A**,**B**) The effect of TGF-β1-reduced smad7 expression in rat hepatic stellate cells (HSCs). Cells were treated with TGF-β1 (5 ng/mL) for 0, 6, 12, and 24 h or TGF-β1 (1, 2, and 5 ng/mL) for 24 h, and smad7 expression was analyzed by Western blotting; (**C**,**D**) the effect of PA-induced smad7 expression in rat HSCs. Cells were treated with PA (2 μM) for 0, 6, 12, and 24 h or PA (0.5, 1, and 2 μM) for 24 h, and smad7 expression was analyzed by Western blotting; (**E**) the effect of PA on TGF-β1-reduced smad7 expression in rat HSCs. Cells were pretreated with 0.5, 1, and 2 μM PA for 1 h, and then stimulated with TGF-β1 (5 ng/mL) for 24 h. The total protein extracted from cells was subjected to Western blotting to determine smad7 expression. Protein bands were imaged using densitometry and analyzed using ImageJ software. The relative expression levels of target proteins were normalized using β-actin as an internal control. The results are presented as the means ± SD of three independent experiments. # Significantly different from the control (*P* < 0.01). * Significantly different from the TGF-β1-treated group (*p* < 0.01).

In contrast, smad7 acts as a smad inhibitor by inhibiting the TGF-β1-dependent SMAD signaling pathway by interfering with smad2 and smad3 phosphorylation via TβRI [29,30]. To examine the effects of PA on TGF-β1-reduced smad7 expression, we first determined the timing and concentration of smad7 in HSC-T6 cells. smad7 expression was inhibited by TGF-β1 treatment in a time- and concentration-dependent manner (Figure 4A,B). However, PA increased smad7 expression in a time- and concentration-dependent manner (Figure 4C,D). Furthermore, PA restored TGF-β1-reduced smad7 expression in a concentration-dependent manner (Figure 4E). Taken together, these results suggested that PA promoted smad7 expression by blocking smad2 and smad3 phosphorylation, inhibiting the TGF-β-dependent SMAD signaling pathway involved in HSC-T6 cell activation.

### 3.4. PA Reduces TGF-β1-Induced HSCs Activation by Upregulation of PPARγ

**Figure 5:**
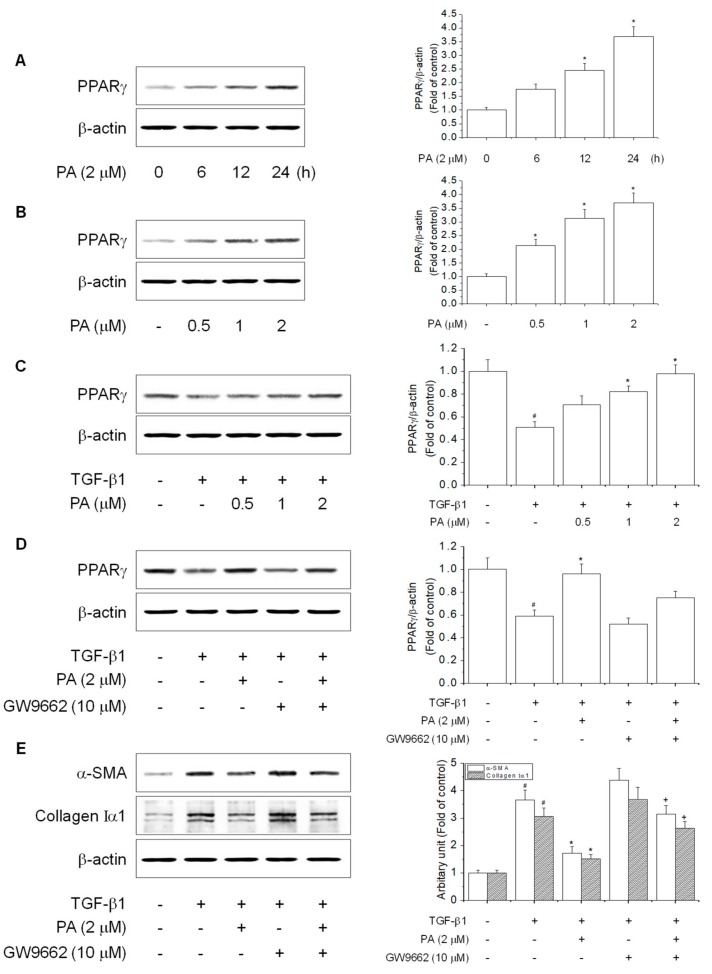
The effects of PA on TGF-β1-reduced PPARγ expression in HSC-T6 cells. (**A**,**B**) The effect of PA-induced PPARγ expression in rat hepatic stellate cells (HSCs). Cells were treated with PA (2 μM) for 0, 6, 12, and 24 h or PA (0.5, 1, and 2 μM) for 24 h, and PPARγ expression was analyzed by Western blotting. The effect of PA on TGF-β1-reduced PPARγ expression in rat HSCs; (**C**) cells were pretreated with various concentrations of PA (0.5, 1, and 2 μM) for 1 h, and then stimulated with TGF-β1 (5 ng/mL) for 24 h. Total protein extracted from cells was subjected to Western blotting to determine PPARγ expression; (**D**,**E**) cells were treated with TGF-β1 (5 ng/mL) for 24 h in the presence of PA and/or GW9662 (10 μM). Total protein extracted from cells was subjected to Western blotting to determine the expression of PPARγ and α-SMA and collagen Iα1. Protein bands were imaged using densitometry and analyzed using ImageJ software. The relative expression levels of target proteins were normalized using β-actin as an internal control. The results are presented as the means ± SD of three independent experiments. # Significantly different from the control (*p* < 0.01). * Significantly different from the TGF-β1-treated group (*p* < 0.01). ^+^ Significantly different from the PA-treated group (*p* < 0.01).

PPARγ is a key molecular switch that regulates HSCs activation and phenotypic alterations to maintain a quiescent HSCs phase that includes the prevention of the TGF-β1 signal, suppression of α-SMA, loss of type I collagen, and reduction of cell proliferation [11,31]. Therefore, PPARγ plays an important role in reducing and preventing liver fibrosis via inhibition of HSCs activation. PPARγ is highly expressed in quiescent HSCs, while its expression is decreased during HSCs activation [32]. However, the effect of PA on the mechanism of PPARγ has not been clearly established during HSCs activation. PA treatment resulted in increased PPARγ expression in a time- and concentration-dependent manner (Figure 5A,B) and restored TGF-β1-reduced PPARγ expression in a concentration-dependent manner (Figure 5C). Together, the results indicated that PA reduced TGF-β1-induced HSCs activation by the activation of PPARγ. 

Studies have reported that the PPARγ antagonist, GW9662, abolished the inhibitory effect of HSCs activation via suppression of α-SMA and type I collagen expression [10]. We confirmed the inhibitory effect of PA on TGF-β1-induced HSCs activation, which was correlated with an increase of PPARγ. PA restored TGF-β1-reduced PPARγ expression, which was eliminated by GW9662 (Figure 5D). Furthermore, PA suppression of TGF-β1-induced expression of α-SMA and collagen Iα1 was abrogated by GW9662 (Figure 5E). Together, the results demonstrated that PA suppressed TGF-β1-induced HSCs activation by upregulation of PPARγ in HSC-T6 cells

### 3.5. PA Reduces TGF-β1-Induced HSCs Activation by Blocking the SMAD-Independent Signal Pathway

**Figure 6:**
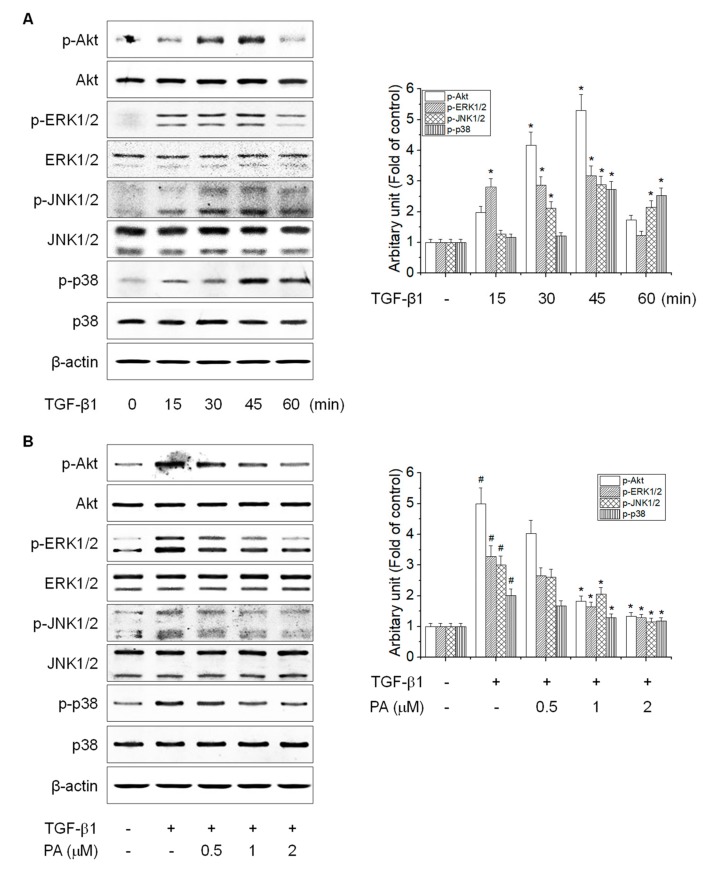
The effects of PA on the TGF-β1-induced Akt and MAPK signal pathways in HSC-T6 cells. (**A**) The effect of TGF-β1-induced Akt and MAPKs phosphorylation in rat hepatic stellate cells (HSCs). HSCs were treated with TGF-β1 (5 ng/mL) for 0, 15, 30, 45, and 60 min, and Akt and MAPKs phosphorylation was analyzed by Western blotting; (**B**) the inhibitory effect of PA on TGF-β1-induced Akt and MAPK phosphorylation in rat HSCs. Cells were pretreated with 0.5, 1, and 2 μM PA for 1 h, and then stimulated with TGF-β1 (5 ng/mL) for 45 min. Total protein extracted from cells was subjected to Western blotting to assess Akt, ERK1/2, JNK1/2, and p38 MAPK phosphorylation. Protein bands were imaged using densitometry and analyzed using ImageJ software. The relative phosphorylation levels of target proteins were normalized using β-actin as an internal control. The results are presented as the means ± SD of three independent experiments. # Significantly different from the control (*p* < 0.01). * Significantly different from the TGF-β1-treated group (*p* < 0.01).

Besides the TGF-β1-dependent SMAD pathway, HSCs are activated by a TGF-β1-independent non-SMAD pathway, which includes Akt and MAPKs [33,34]. Akt and MAPKs are important mediators of signal transduction involved in the activation of HSCs, which can lead to the regulation of cellular growth, differentiation, and proliferation [35,36]. To examine the effects of PA on TGF-β1-induced phosphorylation of Akt and MAPKs, we first determined the timing of TGF-β1-induced Akt and MAPKs phosphorylation in HSC-T6 cells. TGF-β1 induced a significant increase in phosphorylation of Akt, ERK1/2, JNK1/2, and p38 MAPK at 45 min (Figure 6A), which PA diminished in a concentration-dependent manner (Figure 6B). 

**Figure 7:**
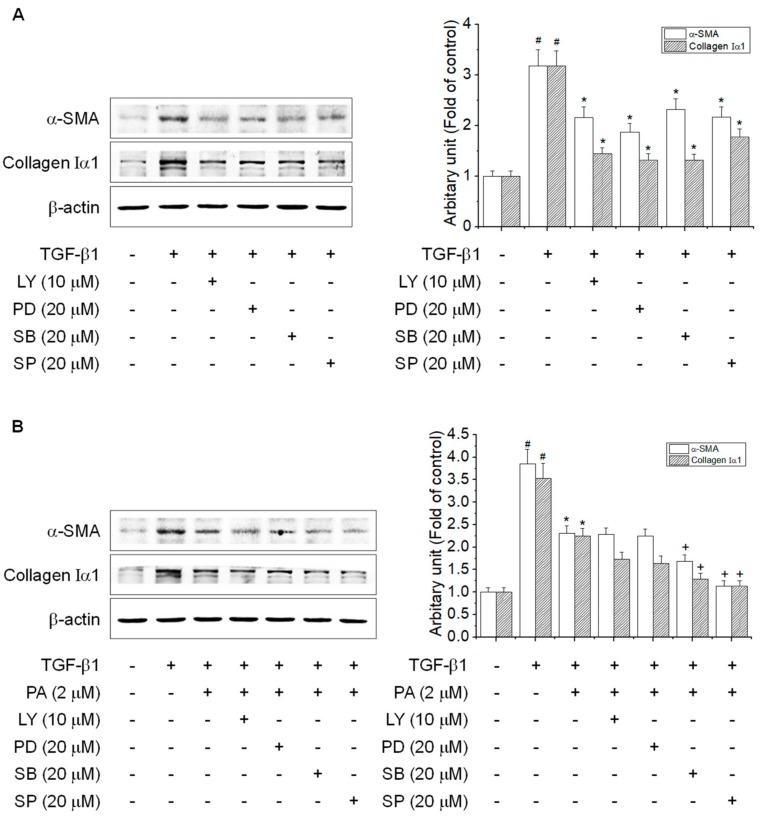
The effects of PA on TGF-β1-induced α-SMA and collagen Iα1 expression via suppression of the Akt and MAPK signal pathways in HSC-T6 cells. (**A**,**B**) The inhibitory effects of PA on TGF-β1-induced α-SMA and collagen Iα1 expression via suppression of Akt and MAPK in rat hepatic stellate cells (HSCs); (**A**) cells were treated with TGF-β1 (5 ng/mL) for 24 h in the presence of LY294002 (LY; 10 μM), PD98059 (PD; 20 μM), SB203580 (SB; 20 μM), or SP600125 (SP; 20 μM); (**B**) cells were treated with TGF-β1 (5 ng/mL) for 24 h in the presence of PA and LY294002 (LY; 10 μM), PD98059 (PD; 20 μM), SB203580 (SB; 20 μM), or SP600125 (SP; 20 μM). Total protein extracted from cells was subjected to Western blotting to determine α-SMA and collagen Iα1 expression. Protein bands were imaged using densitometry and analyzed using ImageJ software. The relative expression levels of target proteins were normalized using β-actin as an internal control. The results are presented as the means ± SD of three independent experiments. # Significantly different from the control (*p* < 0.01). * Significantly different from the TGF-β1-treated group (*p* < 0.01). ^+^ Significantly different from the PA-treated group (*p* < 0.01).

Although PA inhibited the phosphorylation of Akt, ERK1/2, JNK1/2, and p38 MAPK, it is necessary to study whether the inhibitory effect of PA on Akt and MAPKs is related the HSCs activation. We confirmed that Akt and MAPKs inhibitors suppressed the TGF-β1-induced expression of α-SMA and collagen Iα1 (Figure 7A). To study which kinases are involved in the inhibitory effect of PA on HSCs activation, we tested the combined treatment with PA and each inhibitor in cells. As shown in Figure 7B, Akt and ERK1/2 inhibitor did not affect PA-mediated inhibition of α-SMA and collagen Iα1 induced by TGF-β1. These results indicated that Akt and ERK1/2 pathways may involve in the inhibitory effect of PA on TGF-β1-induced expression of α-SMA and collagen Iα1 as a non-SMAD pathway.

## 4. Discussion

Liver disease is a major progressive disorder that negatively impacts human health worldwide [37]. Reports have suggested various drugs as protective or inhibitory agents of hepatofibrogenesis, the molecular mechanisms of which have been extensively studied, both in vitro and in vivo [38]. Our previous studies suggested that aqueous extract and saponin derived from Platycodi radix ameliorated various liver diseases, including ethanol-induced alcoholic liver diseases, high-fat diet-induced non-alcoholic fatty liver disease, nonalcoholic steatohepatitis, and carbon tetrachloride- and dimethylnitrosamine-induced liver fibrosis [23,24,25,26,39]. However, it has been difficult to determine the SMAD-related mechanisms involved in the inhibitory effects of PA, the active component of Platycodi radix-derived saponin, on liver fibrosis during hepatic fibrogenesis. Therefore, we investigated the inhibitory mechanism of PA during HSC activation via SMAD-dependent and SMAD-independent signal pathways by TGF-β1 in rat HSC-T6 cells.

Liver fibrosis is the result of chronic liver disease, which involves a complex process associated with excessive hepatocellular damage. The main causes of liver fibrosis, which is characterized by excessive deposition of extracellular matrix (ECM) proteins, are chronic alcoholic ingestion, obesity, and viral hepatitis infection [40,41]. Quiescent HSCs are activated and transdifferentiated into cells such as activated myofibroblasts to express SMA and various types of collagen proteins, resulting in ECM deposition during hepatic fibrogenesis. Constant accumulation of collagen causes the liver and vascular structure to be distorted, leading to liver dysfunction, scarring, and liver fibrosis [42]. Therefore, the activation of HSCs during liver fibrosis is an important marker of the initiation of chronic liver disease.

TGF-β1, which regulates cell proliferation, differentiation, and ECM formation, is a potent activator of HSCs. When activating HSCs, TGF-β1 promotes cell proliferation via increased expression of α-SMA and collagen I, leading to excess ECM production and liver fibrosis [30]. Therefore, blocking TGF-β1 activity is an effective and logical strategy for preventing or inhibiting liver fibrosis. Our previous studies showed that aqueous extract and saponin derived from Platycodi radix inhibited carbon tetrachloride-, dimethylnitrosamine-, and high-fat diet-induced expression of α-SMA and type I collagen in an animal model [23,24,26]. In the present study, we confirmed that PA, the active component of Platycodi radix-derived saponin, inhibited TGF-β1-induced cell proliferation via suppression of α-SMA and collagen Ia1 in a concentration-dependent manner. Notably, PA treatment inhibited α-SMA and collagen Iα1 expression increased by TGF-β1, and simultaneously decreased mRNA and protein expression. These results indicated that inhibition of HSCs by PA is necessary for the inhibition of TGF-β1-regulated transcription and translation.

The molecular mechanism of HSCs activation has been shown to involve SMAD-dependent and SMAD-independent signal pathways [43,44]. Importantly, the SMAD pathways have been extensively characterized as the main intracellular signaling pathways of TGF-β1. TGF-β1 binds to TβRI, followed by subsequent phosphorylation, activation, and complexing with TβRII, which results in phosphorylation of smad2 and smad3. The TGF-β1 signal then results in continuous translocation to the nucleus, where it activates HSCs via the upregulation of fibrogenic responses. In contrast, smad7 downregulates the phosphorylation of smad2 and smad3 by inhibition of TβRI to inhibit the combined binding of smad2 and smad3 with the receptors [10]. Our results further showed that PA inhibited TGF-β1-induced SBE4 luciferase activity via suppression of smad2/3 phosphorylation by TβRI and TβRII in a concentration-dependent manner. PA also restored TGF-β1-reduced smad7 expression. Notably, PA treatment strongly increased smad7 expression in a time- and concentration-dependent manner. These results indicated that PA treatment attenuated the TGF-β signal pathway and effectively antagonized the activation of HSCs. PA promoted smad7 expression, blocking smad2 and smad3 phosphorylation, to inhibit the TGF-β-dependent SMAD signaling pathway involved in HSCs activation.

Recent studies have also reported that TGF-β1 stimulates the activation of the Akt and MAPK signaling pathways, which further activate specific transcription factors for specific response elements of profibrogenic markers in HSCs [45,46]. The Akt and MAPK pathways, including ERK1/2, JNK1/2, and p38 MAPK, are responsible for the regulation of many cellular functions, including proliferation and apoptosis, which can lead to activation of HSCs. Inhibition of the Akt and MAPK pathways contributes to suppressing HSCs proliferation. Furthermore, our results showed that TGF-β1 induced Akt and MAPKs phosphorylation in a TGF-β-independent signal pathway. PA treatment inhibited phosphorylation of Akt, ERK1/2, JNK1/2, and p38 MAPK by TGF-β1-stimulation. However, we confirmed that combined treatment with PA and inhibitor of JNK1/2 or p38 MAPK showed a more pronounced inhibition of expression of α-SMA and collagen Iα1 induced by TGF-β1 than treatment with PA alone, but not with the inhibitor of Akt or ERK1/2. These results indicated that Akt and ERK1/2 pathways might be major non-SMAD signal pathways involved in the inhibitory effect of PA on HSCs activation. Nevertheless, further study is needed to clarify how PA regulates Akt and MAPK pathways.

PPARγ, as an important nuclear transcription factor, is involved in inhibiting the activation of HSCs, and HSCs activation suppresses the expression and activity of PPARγ, both in vitro and in vivo. Enhancement of PPARγ reduced cell proliferation via suppression of α-SMA and type I collagen expression by blocking TGF-β1 signal transduction in HSCs [31]. In addition, several natural drugs have been shown to increase PPARγ-inhibited profibrogenic marker expression stimulated by TGF-β1 in HSCs [12]. Our results showed that PA treatment significantly increased PPARγ expression in a time- and concentration-dependent manner. Moreover, we confirmed that the activation of PPARγ might be involved in the inhibition of α-SMA and collagen Iα1 expression by PA in TGF-β1-activated HSCs. These results indicated that PA reduced TGF-β1-induced HSC activation through enhancement of PPARγ in HSC-T6 cells.

## 5. Conclusions

**Figure 8:**
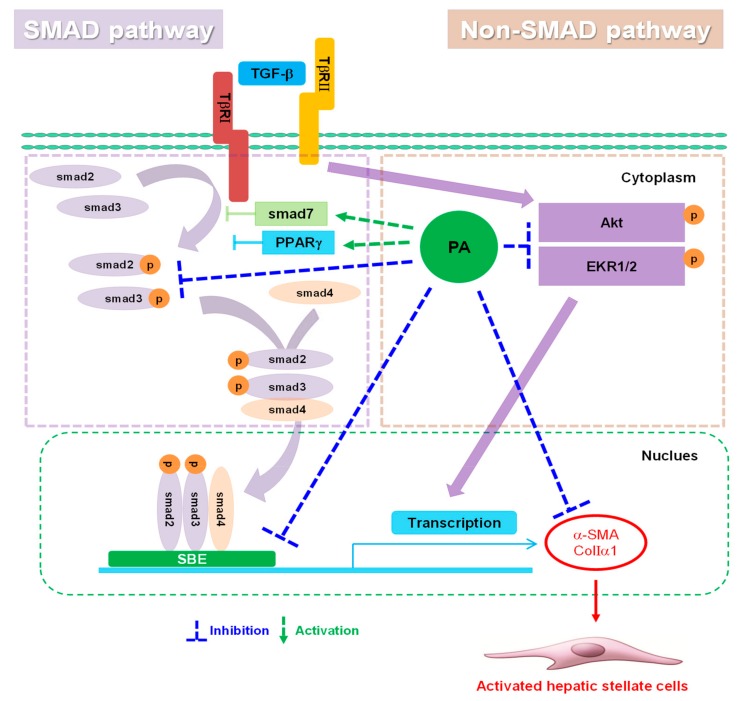
Schematic diagram illustrating the mechanism by which platyconic acid A (PA) inhibits hepatic stellate cell (HSC) activation. PA significantly suppressed α-SMA and collagen Iα1 expression in rat HSCs by blocking the transforming growth factor (TGF)-β1-dependent SMAD signaling pathways and TGF-β1-independent non-SMAD signaling pathways. The mechanism of the TGF-β1-dependent SMAD signaling pathway involved attenuation of smad2/3 by enhancing smad7 and the PPARγ. The mechanism of the TGF-β1-independent non-SMAD signaling pathway involved inhibition of Akt and ERK1/2. Therefore, PA, the active component of Platycodi radix-derived saponin, is a useful chemotherapeutic agent that may prevent HSCs activation.

In conclusion, the present study provided evidence for the inhibitory effect of platyconic acid A (PA), the active component of Platycodi radix-derived saponin, in rat HSCs. PA inhibited TGF-β1-induced HSCs activation through the suppression of SMAD-dependent and SMAD-independent signal transduction pathways (Figure 8). Overall, our results suggest that PA represents a potential candidate for the development of novel chemotherapeutic agents that may contribute to the prevention of liver fibrosis.

## Figures and Tables

**Table 1:** Primer sequences used for the real-time PCR analysis.

Gene	Forward	Reverse
α-SMA	CATCACCAACTGGGACGACA	TCCGTTAGCAAGGTCGGATG
ColIa1	AATCAGCTGGAGTTTCCGTG	TTGGAAACCTTGAGGACCAGG
GAPDH	GGCAAGTTCAATGGCACAGT	AAGGTGGAGGAATGGGAGTT

## Abbreviations

α-SMA	alpha-smooth muscle actin
ECM	extracellular matrix
PA	platyconic acid A
PPARγ	peroxisome proliferator-activated receptor gamma
SBE	smad binding elements
TGF-β	transforming growth factor-β
